# A selective insecticidal protein from *Pseudomonas mosselii* for corn rootworm control

**DOI:** 10.1111/pbi.12806

**Published:** 2017-10-01

**Authors:** Jun‐Zhi Wei, Jessica O'Rear, Ute Schellenberger, Barbara A. Rosen, Young‐Jun Park, Mark J. McDonald, Genhai Zhu, Weiping Xie, Adane Kassa, Lisa Procyk, Claudia Perez Ortega, Jian‐Zhou Zhao, Nasser Yalpani, Virginia C. Crane, Scott H. Diehn, Gary A. Sandahl, Mark E. Nelson, Albert L. Lu, Gusui Wu, Lu Liu

**Affiliations:** ^1^ DuPont Pioneer Hayward CA USA; ^2^ DuPont Pioneer Johnston IA USA; ^3^ Present address: TeneoBio Inc. 1490 O'Brien Drive Menlo Park CA 94025 USA

**Keywords:** western corn rootworm, *Diabrotica*, insecticidal protein, *Pseudomonas*, mode of action

## Abstract

The coleopteran insect western corn rootworm (WCR,* Diabrotica virgifera virgifera*) is an economically important pest in North America and Europe. Transgenic corn plants producing *Bacillus thuringiensis* (*Bt)* insecticidal proteins have been useful against this devastating pest, but evolution of resistance has reduced their efficacy. Here, we report the discovery of a novel insecticidal protein, PIP‐47Aa, from an isolate of *Pseudomonas mosselii*. PIP‐47Aa sequence shows no shared motifs, domains or signatures with other known proteins. Recombinant PIP‐47Aa kills WCR, two other corn rootworm pests (*Diabrotica barberi* and *Diabrotica undecimpunctata howardi*) and two other beetle species (*Diabrotica speciosa* and *Phyllotreta cruciferae*), but it was not toxic to the spotted lady beetle (*Coleomegilla maculata*) or seven species of Lepidoptera and Hemiptera. Transgenic corn plants expressing PIP‐47Aa show significant protection from root damage by WCR. PIP‐47Aa kills a WCR strain resistant to mCry3A and does not share rootworm midgut binding sites with mCry3A or AfIP‐1A/1B from *Alcaligenes* that acts like Cry34Ab1/Cry35Ab1. Our results indicate that PIP‐47Aa is a novel insecticidal protein for controlling the corn rootworm pests.

## Introduction

The western corn rootworm (WCR, *Diabrotica virgifera virgifera*) is one of the most devastating corn pests in Northern America (Gray *et al*., 2009) and Europe (Ciosi *et al*., 2008). The larvae, feeding on corn roots, injure and damage the root system leading to reduction in water and nutrient absorption and plant lodging (Reidell, 1990). In addition, the wounded areas provide opportunities for secondary infestations by root pathogens and other pests. It is estimated that WCR infestations in the United States can cause severe corn yield reduction with a loss of over $1 billion in revenue per year (Sappington *et al*., 2006). Prior to the introduction of transgenic corn with WCR control traits, crop rotation to nonhost crops and pesticide applications were major methods used to control WCR infestations. As WCR is a highly adaptive pest, over time the insects evolved to lay eggs in fields of noncorn crops (such as soybean) (Levine *et al*., 2002) and to become resistant to conventional insecticides (Zhu *et al*., 2001), making these strategies less effective. Furthermore, crop rotation is not always practical in the US Corn Belt and chemical insecticides can cause environmental concerns (Sappington, 2014). Since 2003, farmers in the United States have widely adopted transgenic corn producing *Bacillus thuringiensis* (*Bt*) insecticidal proteins to control WCR (Sanahuja *et al*., 2011). This significantly reduces conventional insecticide usage and increases economic benefits to farmers (Fernandez‐Cornejo *et al*., 2014). To date, *Bt*‐derived proteins Cry3Bb1, mCry3A, eCry3.1Ab and Cry34Ab1/Cry35Ab1 have been deployed in commercial corn products to control WCR (Moellenbeck *et al*., 2001; Narva *et al*., 2013; Rice, 2004). However, after a decade of application of this transgenic strategy, varying levels of field‐evolved resistance have been documented for Cry3Bb1 (Gassmann *et al*., 2011) and Cry34Ab1/Cry35Ab1 (Gassmann *et al*., 2016). Rootworms resistant to Cry3Bb1 have also been shown to be cross‐resistant to related proteins including mCry3A and eCry3.1Ab (Gassmann *et al*., 2014; Jakka *et al*., 2016). The emergence of resistant WCR could be due to a combination of factors, including fast product adoption, shared sites of action of insecticidal proteins, lack of high‐dose trait, lack of compliance for refuge planting and WCR's strong adaptability (Gassmann, 2016; Tabashnik and Gould, 2012).

To enhance trait durability, insecticidal proteins with novel sites of action are needed for development of the next generation of transgenic corn rootworm control traits (Gassmann, 2016). New approaches such as disabling WCR genes by RNA interference through *in‐planta* expression of double‐stranded RNAs (Baum *et al*., 2007; Fishilevich *et al*., 2016; Hu *et al*., 2016) and expressing non‐*Bt* insecticidal proteins in corn (Sampson *et al*., 2017; Schellenberger *et al*., 2016) demonstrate the potential to overcome insect resistance to current commercial traits. Pyramiding multiple new insecticidal proteins with distinct midgut target sites is also essential for enhancing trait durability. Here, we report the discovery of a novel insecticidal protein, designated as PIP‐47Aa, from an isolate of *Pseudomonas mosselii*. Our data indicate that PIP‐47Aa is toxic to three corn rootworm species. It is capable of controlling a WCR strain resistant to mCry3A and does not share receptor binding sites with mCry3A or AfIP‐1A/1B from *Alcaligenes* that acts like Cry34Ab1/Cry35Ab1, currently deployed in commercial corn products. Therefore, PIP‐47Aa holds promise as part of a new rootworm control trait solution in the future.

## Results

### Cell lysate of a *Pseudomonas mosselii* isolate shows strong activity against WCR

We isolated bacterial strains from soil samples collected from fields in the USA. Soluble proteins extracted from cell cultures of individual isolates were mixed with an artificial insect diet and fed to WCR larvae. Isolate SST62E1 consistently showed strong insecticidal activity by killing or severely stunting WCR larvae in the bioassays. The sample lost its insecticidal activity when treated with proteinase K and heat, indicating the active components are proteinaceous. A BLAST search of the SST62E1 16S rDNA sequence against the NCBI database identified the strain as *Pseudomonas mosselii*. The genome of SST62E1 was sequenced, and ORFs were predicted and annotated.

### PIP‐47Aa is a novel insecticidal protein

**Figure 1:**
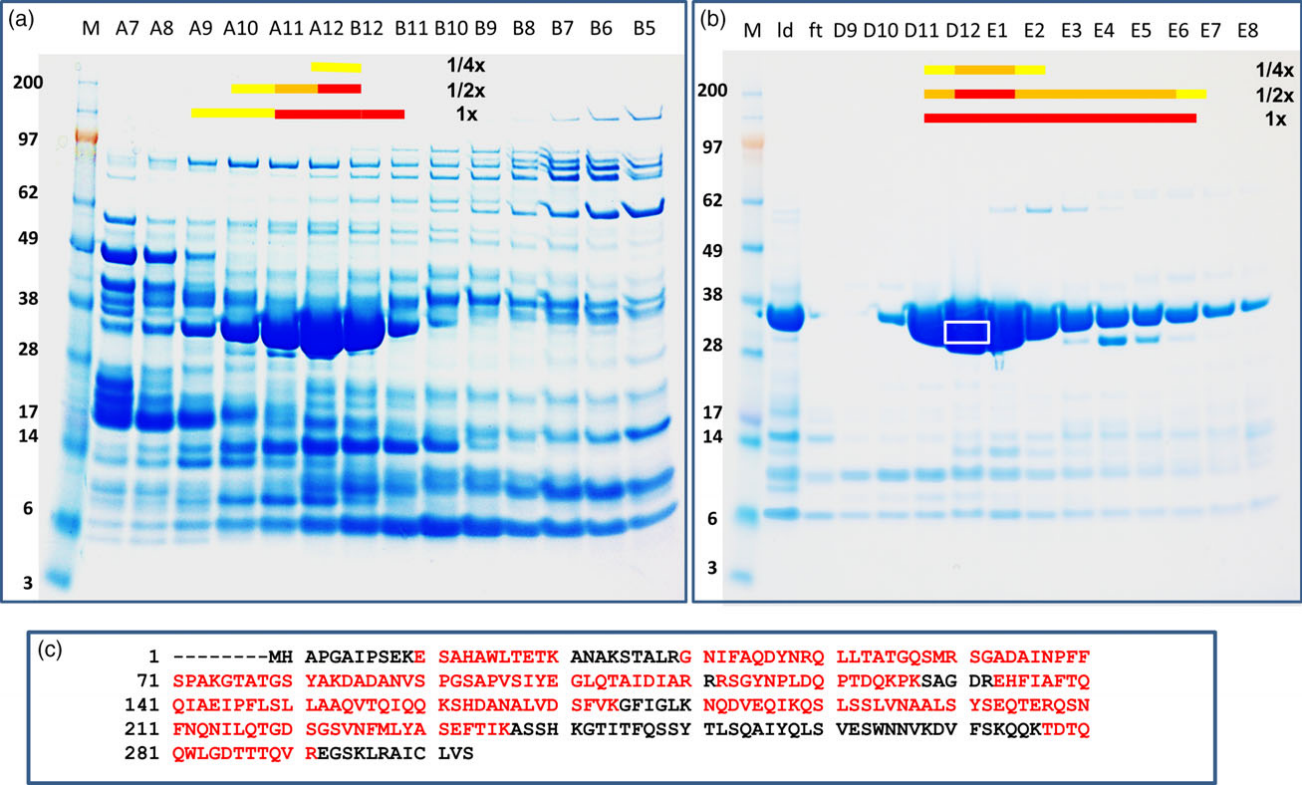
Insecticidal protein purification from isolate SST62E1. (a) SP column separation and bioassay results. (b) Mono Q column separation and bioassay results. (c) Peptide coverage (residues in red) of candidate protein. M, molecular size markers; ld, load; ft, flow through; other numbers are fraction numbers. Red, orange and yellow bars are indicating killing, severe stunting and stunting, respectively. 1×, 1/2× and 1/4× are dilutions of samples in bioassay. White box represents area excised from the gel for in‐gel digestion by trypsin.

Multiple chromatography steps for protein separation were conducted with different purification matrices. After each separation, fractions were subjected to insect diet feeding bioassays. The active fractions were pooled and advanced to further enrichment steps. Chromatography fractions with high insecticidal activity were analysed on SDS–PAGE gels; candidate protein bands were excised, and in‐gel digested with trypsin (Figure 1a,1b). The resultant peptides were analysed by nanoscale liquid chromatography–tandem mass spectrometry (LC‐MS/MS). Database searches against our DuPont Pioneer proprietary genome database with Mascot (Matrix Science Ltd., London, UK) showed a match of a protein band with high peptide coverage to a predicted ORF from the SST62E1 genome sequence (Figure 1c). The candidate gene was designated as *PIP‐47Aa* (*Pseudomonas* Insecticidal Protein, GenBank accession number KY982916). The predicted PIP‐47Aa protein is composed of 295 amino acids with a deduced molecular mass of 32.1 kDa and a pI of 7.15. Queries of the DNA and protein sequences against various databases did not reveal homology to known proteins or matches to Pfam domains or signature peptides, indicating it is a novel protein. The DNA of *PIP‐47Aa* was amplified with PCR from the SST62E1 genomic DNA and subcloned into pCOLD™‐1 plasmid, which was then transformed into *Escherichia coli* competent cells (BL21‐DE3). The purified PIP‐47Aa recombinant protein from *E. coli* showed killing activity on WCR larvae in bioassay, confirming the insecticidal activity of PIP‐47Aa.

### PIP‐47Aa shows highly selective activity against coleopteran insect pests

Dose response assays were carried out with purified PIP‐47Aa protein against WCR. Ten concentrations, starting at 200 μg/mL with serial 2× dilutions and >60 neonates per concentration, were tested. Concentrations killing 50% of larvae tested (LC_50_) and inhibiting growth of 50% of larvae tested (IC_50_) were estimated. The LC_50_ of PIP‐47Aa on WCR was 52.45 μg/mL, with 95% fiducial limit (FL) of 45.68–59.48 μg/mL. The IC_50_ was 20.93 μg/mL, with 95% FL of 16.70–24.86 μg/mL (Table 1). To test the efficacy of PIP‐47Aa on other corn rootworm species, purified PIP‐47Aa protein was assayed against neonates of northern corn rootworm (NCR*, Diabrotica barberi*) and southern corn rootworm (SCR, *Diabrotica undecimpunctata howardi*). The LC_50_ and the IC_50_ of PIP‐47Aa on NCR were 122.03 μg/mL and 10.74 μg/mL, while the LC_50_ and the IC_50_ on SCR were 255.30 μg/mL and 59.10 μg/mL, respectively (Table 1). To further assess the activity spectrum of PIP‐47Aa, multiple insect species of Coleoptera, Lepidoptera and Hemiptera were tested with purified PIP‐47Aa protein (Table 2). Relative to results with WCR, the IC_50_ for San Antonio beetle (*Diabrotica speciosa*) was 7.76‐fold higher and the LC_50_ for Crucifer flea beetle (*Phyllotreta cruciferae*) was 4.95‐fold higher, respectively. No activity was detected on coleopteran spotted lady beetle (*Coleomegilla maculata*), or selected seven lepidopteran and hemipteran insects at the highest tested concentrations (Table 2). The results indicate that PIP‐47Aa kills beetles from five of six species tested; among those species, it is most potent against three species of corn rootworm.

**Table 1:** Insecticidal activity of PIP‐47Aa against neonate WCR, SCR and NCR

Insect	LC_50_ or IC_50_	μg/mL	Lower 95% FL	Upper 95% FL	Slope ± SE	χ^2^ (df)	*P‐*value	*n*	NR (N)
WCR	LC_50_	52.45	45.68	59.48	3.88 ± 0.43	2.96 (4)	0.57	383	0.02 (64)
	IC_50_	20.93	16.70	24.86	2.92 ± 0.41	6.68 (5)	0.25	444	0.06 (64)
SCR	LC_50_	255.30	213.60	306.30	6.59 ± 0.99	4.28 (3)	0.23	160	0 (32)
	IC_50_	59.10	49.20	70.50	6.74 ± 1.04	1.28 (3)	0.14	159	0 (32)
NCR	LC_50_	122.03	91.20	167.50	2.59 ± 0.30	9.23 (7)	0.24	276	0 (30)
	IC_50_	10.74	7.93	13.97	1.93 ± 0.24	8.32 (5)	0.14	215	0.03 (30)

*n*, number of larvae for dose treatment; NR, natural response as proportion mortality in control treatment; *N*, number of larvae for control treatment.

**Table 2:** Insecticidal activity of purified PIP‐47Aa against various insects

Insect	Highest dose tested (ppm)	Estimated IC_50_ or LC_50_ (ppm)	Effect
Coleoptera			
San Antonio beetle (*Diabrotica speciosa*)	1167	163^a^	Death
Crucifer flea beetle (*Phyllotreta cruciferae*)	500	260^b^	Death
Spotted lady beetle (*Coleomegilla maculata*)	1000	N/A	Inactive
Lepidoptera			
Black cutworm (*Agrotis ipsilon*)	730	N/A	Inactive
Corn earworm (*Helicoverpa zea*)	730	N/A	Inactive
Fall armyworm (*Spodoptera frugiperda*)	730	N/A	Inactive
Soybean looper (*Pseudoplusia includens*)	730	N/A	Inactive
European corn borer (*Ostrinia nubilalis*)	730	N/A	Inactive
Hemiptera			
Lygus bug (*Lygus hesperus*)	300	N/A	Inactive
Southern green stink bug (*Nezara viridula*)	300	N/A	Inactive

a IC_50_.

b LC_50_.

### Close homologs of PIP‐47Aa are also active against WCR

**Figure 2:**
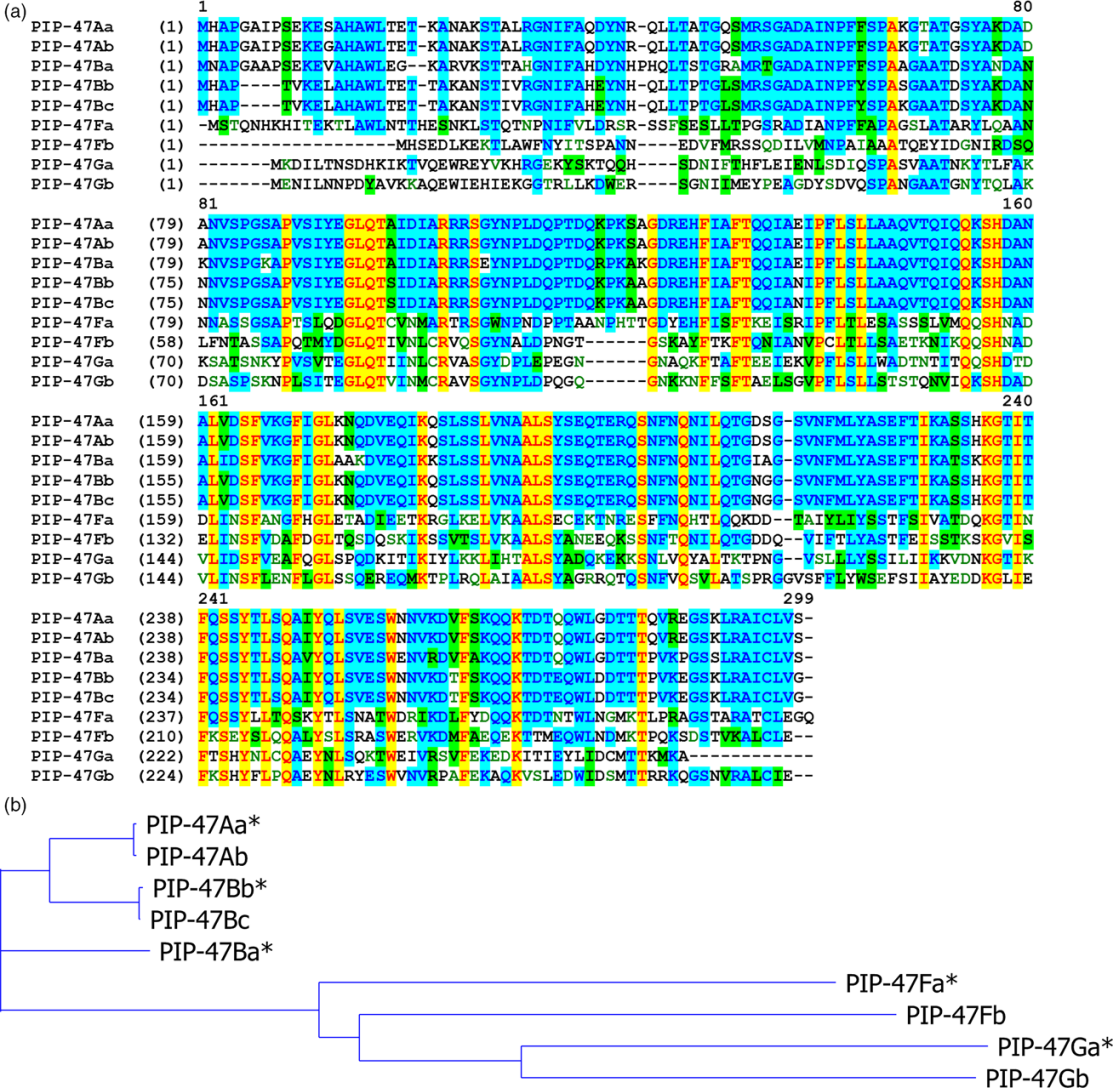
PIP‐47Aa protein homologs. (a) Alignment of PIP‐47Aa and homolog sequences using Vector NTI. Yellow – identical amino acids; blue and green – conserved amino acids. (b) Phylogenetic tree of PIP‐47 homolog sequences generated with Vector NTI. * Indicates homologs that were subcloned into *Escherichia coli* and tested for insecticidal activity.

A BLASTp search against both public and DuPont Pioneer internal databases revealed multiple sequence homologs to PIP‐47Aa. We developed a naming system to classify these sequences based on their percent identities to the PIP‐47Aa protein. For example, PIP‐47A, B, C, D, E, F or G represents homologs with protein sequence identity to PIP‐47Aa as ≥90%, ≥80%, ≥70%, ≥60%, ≥50%, ≥40%, or ≥30%, respectively. A second lower case letter is added to differentiate members in each subgroup such as PIP‐47Ba, PIP‐47Bb or PIP‐47Bc. Homologs from the A and B subgroups are all found in *Pseudomonas* species, while most of the distant homologs are from other bacteria, such as *Photorhadbus*,* Klebsiella*,* Vibrio* and *Raoultella* (Table S1). Figure 2 shows the protein sequence alignment of PIP‐47 homologs and the corresponding phylogenetic tree. A detailed identity table can be found in Table S2. Representative homologs (*PIP‐47Ba*,* PIP‐47Bb*,* PIP‐47Fa* and *PIP‐47Ga*) were subcloned into *E. coli* expression vectors, and the recombinant proteins were assayed against WCR for toxicity. PIP‐47Ba and PIP‐47Bb showed insecticidal activity that was comparable to the activity level of PIP‐47Aa, while PIP‐47Fa and PIP‐47Ga showed no appreciable activity at the highest assayed dosages (730 μg/mL for PIP‐47Fa and 320 μg/mL for PIP‐47Ga).

### Transgenic corn plants expressing PIP‐47Aa exhibit reduction in root injury by WCR

**Figure 3:**
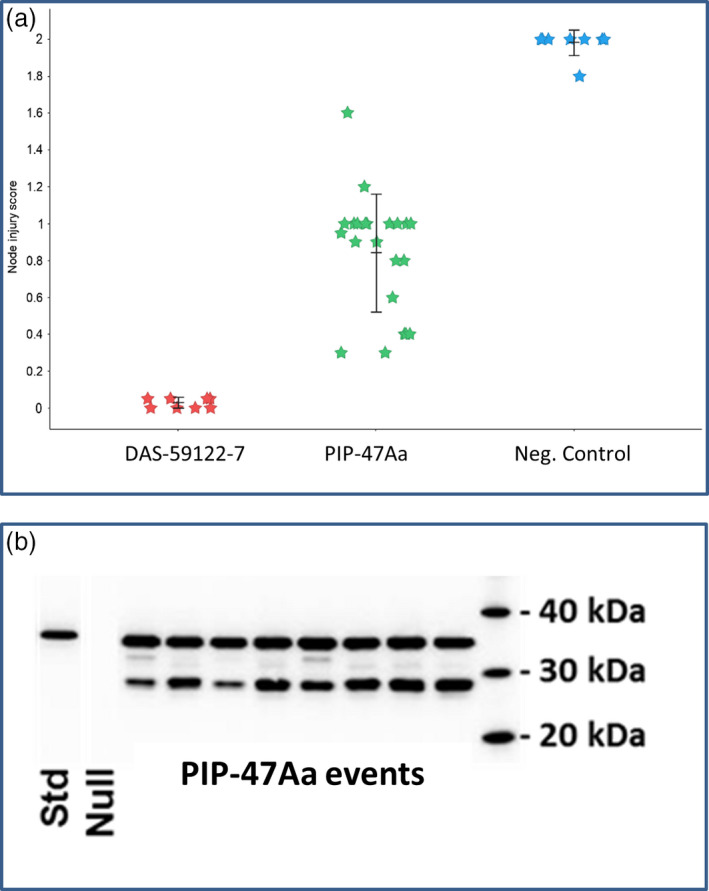
Efficacy of PIP‐47 against WCR and protein accumulation in maize roots. (a) Root protection from WCR injury by transgenic expression of PIP‐47Aa in T0 corn plants grown under glasshouse conditions. Root node‐injury scores from ZmPIP‐47Aa events (green, *n *= 22) compared to nontransgenic corn PHR03 (negative control) plants (blue, *n* = 8) and the commercial corn line DAS‐59122‐7 expressing Cry34Ab1/35Ab1 plants (red, *n* = 8). Bars indicate means and standard errors. (b) Western blot analysis using anti‐PIP‐47Aa antibody.

To test whether PIP‐47Aa can provide root protection, T0 events of transgenic corn expressing a plastid‐targeted PIP‐47Aa under the control of an enhanced root promoter (3XMMV ENH‐Sb‐RCc3 PRO) were evaluated against WCR. Root damage assessment was performed using the Iowa State University 0‐3 Node Injury Scale (Oleson *et al*., 2005). When infested with WCR, nontransgenic negative control plants showed substantial root damage with a mean node‐injury score of 1.98 ± 0.09 (*n* = 8), while positive control plants, DAS 59122‐7 expressing Cry34Ab1/Cry35Ab1, showed strong root protection with a mean node‐injury score of 0.03 ± 0.09 (*n* = 8). Single‐copy transgenic events expressing PIP‐47Aa protein provided substantial root protection from feeding injury with a mean node‐injury score of 0.84 ± 0.05 (*n* = 22) which was statistically different from the negative controls (*DF *= 2, *F*‐value* *= *121.6, P*‐value < 0.001). Several individual events showed root node‐injury scores below 0.5 (Figure 3a), which is the economic threshold, indicating the potential of PIP‐47Aa for rootworm control. PIP‐47Aa protein was detected by Western blot analysis in root tissue from these transgenic plants, although it appears that some of the PIP‐47Aa protein was processed by plant proteases, as observed on SDS–PAGE (Figure 3b).

### PIP‐47Aa kills WCR strain resistant to mCry3A protein

To test the ability of PIP‐47Aa for controlling a WCR strain resistant to mCry3A protein, PIP‐47Aa protein was assayed against an mCry3A‐resistant strain (Zhao *et al*., 2016) and a susceptible laboratory strain. mCry3A is an engineered Cry3A toxin with insecticidal activity against WCR (Walters *et al*., 2008). The results of this testing indicate that the activity of PIP‐47Aa protein is comparable between mCry3A‐resistant and mCry3A‐sensitive WCRs as similar LC_50_ and IC_50_ values observed against both strains (Table 3). The calculated resistance ratio (RR) for PIP‐47Aa is about 1, while the RR for mCry3A protein is 59 against the mCry3A‐resistant colony. These results suggest that there is no cross‐resistance between PIP‐47Aa and mCry3A.

**Table 3:** Insecticidal activity of PIP‐47Aa on WCR strains susceptible or resistant to mCry3A

WCR strain to mCry3A	Data	μg/mL, 3d	95% FL	Slope (SE)	Resistance ratio (RR^a^)
Susceptible	LC_50_	66.00	4.05–125.50	1.97 (0.83)	
	IC_50_	15.24	10.38–19.24	5.94 (1.89)	
Resistant	LC_50_	47.48	31.30–63.10	2.77 (0.55)	0.72
	IC_50_	12.32	8.92–15.59	3.80 (0.81)	0.81

a RR = 59‐fold to mCry3A based on IC50 (290.2/4.93 μg/mL).

### PIP‐47Aa binds specifically to WCR midgut brush border membrane vesicles (BBMVs)

**Figure 4:**
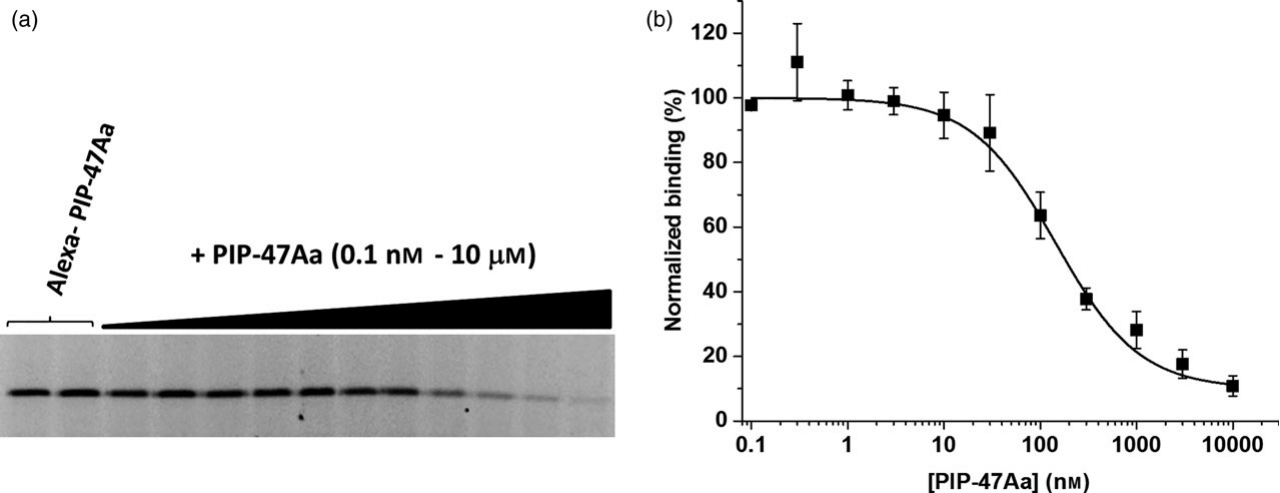
Specific binding of PIP‐47Aa to WCR BBMVs demonstrated by homologous competition. (a) Representative gel image of the homologous competition of Alexa‐labelled PIP‐47Aa by unlabelled PIP‐47Aa. As the concentration of unlabelled PIP‐47Aa is increased, the level of fluorescence decreases reflecting increased occupancy of binding sites by unlabelled protein preventing binding of Alexa‐labelled protein. (b) The graph reflects the average of the densitometry values determined for the fluorescence intensity captured from the gel image displayed as the percentage of total binding (in absence of competitor) versus the concentration of unlabelled PIP‐47Aa. The solid line reflects the fit of the data to a logistic equation to estimate the EC_50_ value which was 145 nM.

To demonstrate specific binding and evaluate apparent affinity, WCR BBMVs (30 μg) were incubated with Alexa‐labelled PIP‐47Aa (1 nM) in binding buffer in the absence or presence of increasing concentrations of unlabelled PIP‐47Aa. The apparent affinity (EC_50_) of PIP‐47Aa for WCR BBMVs was approximately 145 nM (Figure 4). This was defined as the concentration of unlabelled protein that was needed to reduce the binding of Alexa‐labelled PIP‐47Aa by 50%. The EC_50_ value indicates that PIP‐47Aa interacts with specific receptors in WCR midgut membranes with reasonably good affinity.

### PIP‐47Aa does not share binding sites with Cry3A or AfIP‐1A/1B

**Figure 5:**
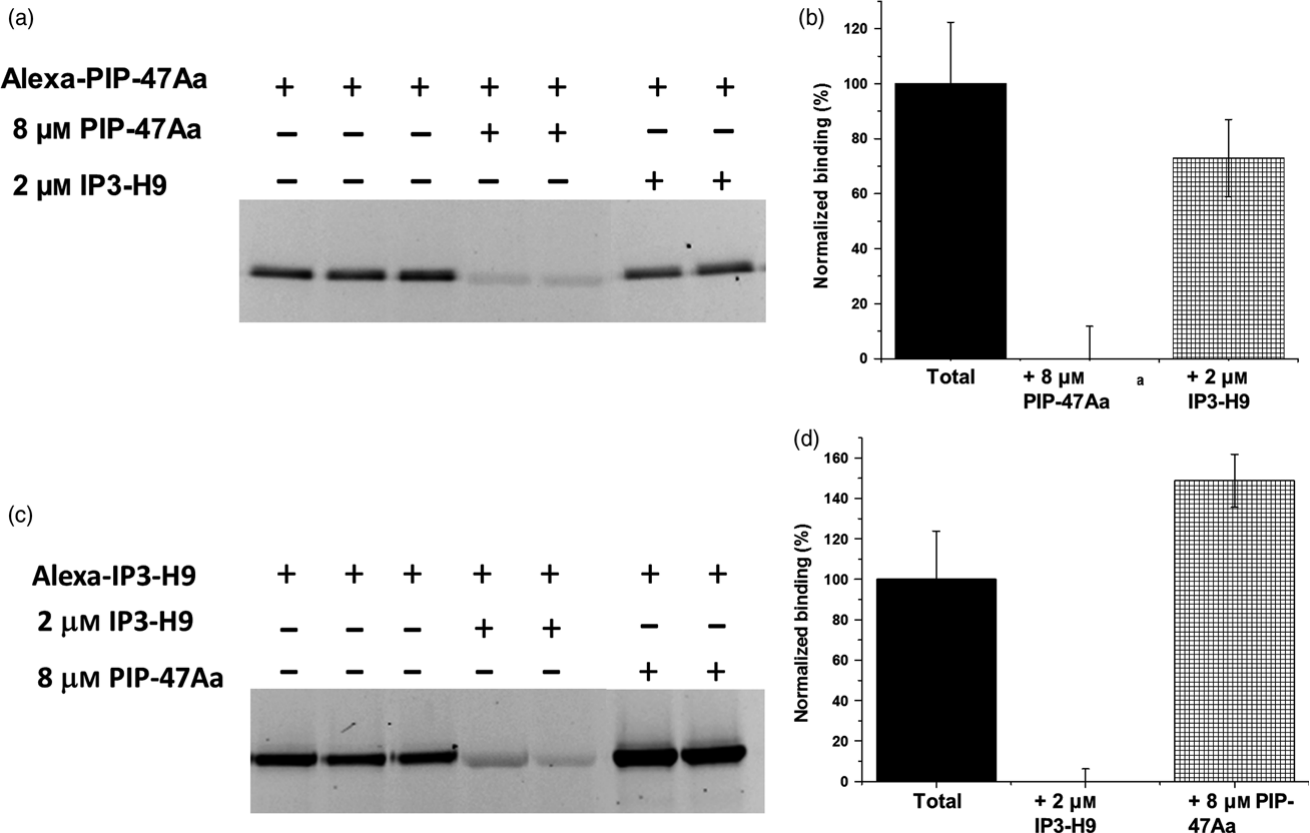
Heterologous competition between PIP‐47Aa and Cry3A variant, IP3‐H9. (a) Representative gel shows that binding of Alexa‐PIP‐47Aa (2 nM) was not affected by a saturating concentration (2 μM) of IP3‐H9. (b) A bar graph depicting the averaged densitometry values with standard error bars determined from gel images as shown in panel a. No significant reduction in Alexa‐PIP‐47Aa binding was observed during heterologous competition. (c) Representative gel showing the reciprocal competition of Alexa‐IP3‐H9 (5 nM) by a saturating concentration (8 μM) of PIP‐47Aa. (d) A bar graph depicting the averaged densitometry values with standard error bars determined from gel images as shown in panel c. No significant reduction in Alexa‐IP3‐H9 binding was observed during heterologous competition.

The ability of PIP‐47Aa protein to kill WCR resistant to mCry3A suggests that both proteins likely recognize different receptor binding site(s) on WCR midgut‐derived BBMVs. We performed a BBMV‐based heterologous competition binding assay between PIP‐47Aa and IP3‐H9, a solubility‐enhanced variant of Cry3Aa, which has been shown to share binding site(s) with mCry3A (Zhao *et al*., 2016). WCR BBMVs (20 μg) were incubated with Alexa‐labelled PIP‐47Aa (2 nM) in binding buffer in the absence or presence of a saturating concentration of unlabelled IP3‐H9 (2 μM). No reduction in fluorescent signal of Alexa‐labelled PIP‐47Aa was observed (Figure 5a,5b). A reciprocal competition assay performed using WCR BBMVs (5 μg), Alexa‐labelled IP3‐H9 (5 nM) and a saturating concentration of unlabelled PIP‐47Aa (8 μM) also did not show a reduction in fluorescent signal of Alexa‐labelled IP3‐H9 (Figure 5c,5d). These results confirm that PIP‐47Aa does not share the same receptor binding site(s) utilized by mCry3A and is consistent with the results of the cross‐resistance testing with mCry3A‐resistant WCR.

**Figure 6:**
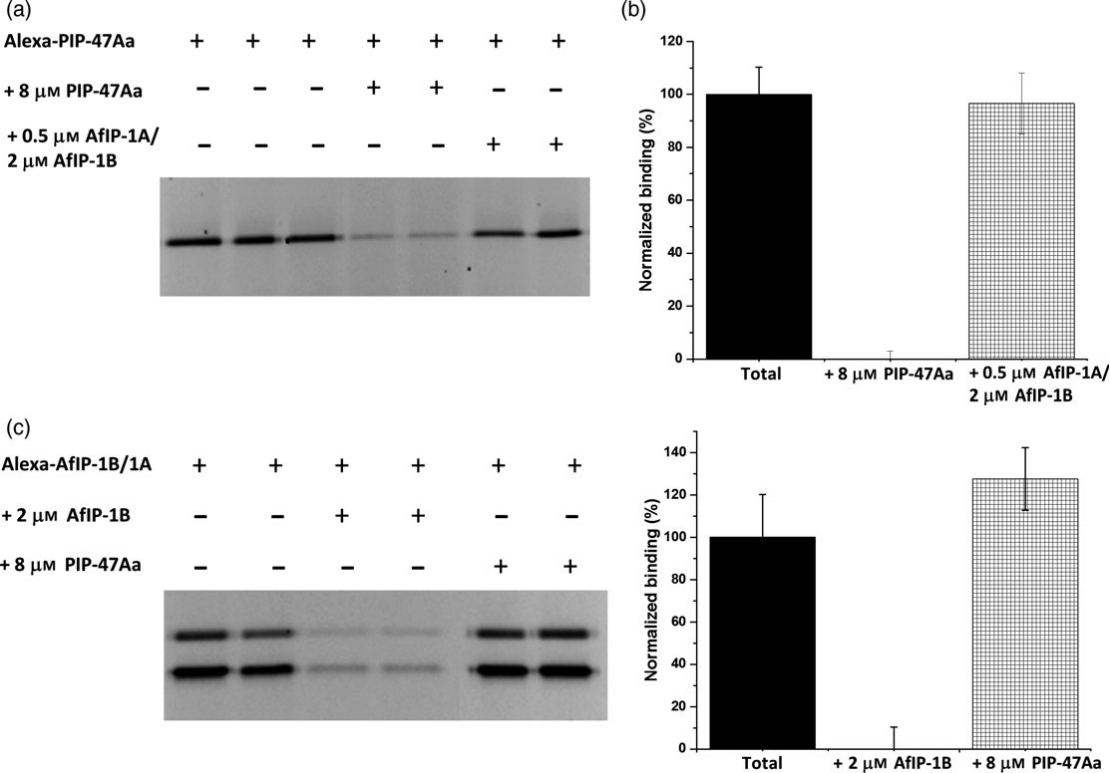
Heterologous competition between PIP‐47Aa and AfIP‐1A/AfIP‐1B to test for cross‐resistance to Cry34/Cry35. (a) A representative gel that depicts the specific binding of Alexa‐PIP‐47Aa (2 nM) and lack of competition by a saturating concentration of a mixture of AfIP‐1A (0.5 μm) and AfIP‐1B (2 μM). (b) A bar graph depicting the averaged densitometry values with standard error bars determined from gel images as shown in panel a. No significant reduction in Alexa‐PIP‐47Aa binding was observed during heterologous competition. Note that AfIP‐1B exists as an N‐ and C‐terminal fragment during binding, so densitometry values for both fragments were analysed to calculate the averaged values. (c) A representative gel depicting the specific binding and reciprocal competition when Alexa‐AfIP‐1B (10 nM) along with AfIP‐1A (500 nM) was incubated in the presence of a saturating concentration (8 μM) of PIP‐47Aa. (d) A bar graph depicting the averaged densitometry values with standard error bars determined from gel images as shown in panel c. No significant reduction in Alexa‐AfIP‐1B binding was observed during heterologous competition.

To test whether PIP‐47Aa shares the same binding site(s) with Cry34Ab1/Cry35Ab1, we used AfIP‐1A/1B from *Alcaligenes*, which shares binding sites with Cry34Ab1/Cry35Ab1 (Yalpani *et al*., 2017). WCR BBMVs (20 μg) were incubated with 2 nM Alexa‐labelled PIP‐47Aa in the binding buffer in the absence or presence of a saturating concentration of an AfIP‐1A and AfIP‐1B mixture (0.5 μM AfIP‐1A and 2 μM AfIP‐1B). Reciprocal heterologous competition experiments were also performed using 10 nM Alexa‐labelled AfIP‐1B along with 0.5 μM AfIP‐1A in the absence or presence of a saturating concentration of unlabelled PIP‐47Aa (8 μM). No reduction in fluorescent signal of either Alexa‐labelled PIP‐47Aa or Alexa‐labelled AfIP‐1B/unlabelled‐AfIP1A was observed (Figure 6). These results indicate that PIP‐47Aa does not share receptor binding site(s) recognized by AfIP‐1A/1B, representing Cry34Ab1/Cry35Ab1. Overall, the data suggest that PIP‐47Aa kills WCR by interacting with receptor binding site(s) different from those used by the two commercial *Bt*‐derived WCR actives.

## Discussion

*Bacillus* bacteria, especially *Bt* strains have been the major source for insecticidal protein discovery for the last several decades. All current commercialized transgenic crops with insect control traits are developed with *Bt*‐derived protein encoding genes (Palma *et al*., 2014; Pardo‐Lopez *et al*., 2013). Although new *Bt* toxins are still being discovered, it is challenging to find new insecticidal proteins that can be easily deployed in commercial products, primarily due to their limited spectrum, potency or lack of unique mode of action compared to existing traits. For WCR control, Cry3 proteins (including Cry3Bb, mCry3A and eCry3.1Ab) and Cry34Ab1/35Ab1 are the only available options that all together represent two distinct modes of actions in current commercialized transgenic corn. Non‐*Bt* bacteria have been screened against various insects. For example, some insect pathogenic bacteria have been explored for potential applications in pest control (Ruiu, 2015). Monalysin, a β‐pore‐forming toxin from *P. entomophila,* showed lethal effects on *Drosophila* (Opota *et al*., 2011). *P. taiwanensis*, a broad‐host‐range entomopathogenic bacterium, exhibited insecticidal activity towards agricultural pests, and its large molecular weight multicomponent toxin complex (Tc) was found to play an essential role in the activity (Chen *et al*., 2014). Recently, novel insecticidal proteins with activity against key crop insect pests have been identified from non‐*Bt* microbes. These include a tiny insecticidal protein (Tip), IPD072Aa from *P. chlororaphis* (Schellenberger *et al*., 2016; Tabashnik, 2016), an insecticidal protein, GNIP1Aa, from *Chromobacterium piscinae* (Sampson *et al*., 2017), and a binary rootworm active AfIP‐1A/1B, from *Alcaligenes* (Yalpani *et al*., 2017). Transgenic corn plants expressing IPD072Aa showed strong efficacy in glasshouse and field tests against WCR. IPD072Aa killed WCR resistant to mCry3A or Cry34Ab1/Cry35Ab1, making it a promising candidate for WCR control (Schellenberger *et al*., 2016). Although AfIP‐1A/1B killed WCR resistant to mCry3A, it was not effective against WCR resistant to Cry34Ab1/Cry35Ab1, which limits its potential applications for WCR control. The presence of a MACPF domain within GNIP1Aa suggests that it may exert its function by pore formation in the insect midgut membrane (Sampson *et al*., 2017). The utility of GNIP1Aa as a rootworm active remains to be determined until information on cross‐resistance to current commercial rootworm actives becomes available.

Here, we describe the discovery and characterization of a unique non‐*Bt* insecticidal protein, PIP‐47Aa, from an isolate of *P. mosselii*. PIP‐47Aa appears to be specific to certain coleopteran insect species, especially to the members of the corn rootworm complex. Furthermore, it is not active against the spotted lady beetle and various lepidopteran and hemipteran insect species. Such high specificity indicates that PIP‐47Aa is a selective toxin. PIP‐47Aa has no shared motifs, domains or signatures to other known proteinaceous toxins, implying that it might function differently compared to existing *Bt*‐derived toxins. Indeed, our data confirmed that PIP‐47Aa is capable of killing WCR resistant to mCry3A and does not share midgut receptor binding site(s) with Cry3A or Cry34Ab1/Cry35Ab1. Moreover, protein domain comparison by Pfam indicates that PIP‐47Aa does not belong to any protein family shared by any of the newly discovered non‐*Bt* actives including GNIP1Aa and IPD072Aa (Tip). It therefore seems unlikely that PIP‐47Aa shares the same receptor binding site(s) with those new actives. Additional experiments are needed to confirm this hypothesis. Evaluation of *PIP‐47Aa* in transgenic corn also demonstrated its ability to protect roots from WCR feeding damage. Although the root node‐injury score is not as low as for corn producing Cry34Ab1/Cry35Ab1, *PIP‐47Aa* holds promise as a new tool to help combat this devastating pest. The discovery of PIP‐47Aa, along with several earlier examples (Sampson *et al*., 2017; Schellenberger *et al*., 2016; Yalpani *et al*., 2017), indicates diverse bacteria other than *Bt* represent a potentially rich source for novel insecticidal proteins. Although the mechanism of insect toxicity of these newly discovered non‐*Bt* proteins awaits further investigation, they share some characteristics with *Bt* proteins. Both types of proteins demonstrate selectivity for only certain insect species. They are orally active and bind specifically to targets located in insect midgut, suggesting a toxic effect through the digestive tract. As we continue to explore and characterize these new insecticidal proteins, it is hopeful that they will become new tools for controlling insect pests.

## Experimental procedures

### Bacterial strain preparation

The bacterial isolation and preparation follow previously described methods (Schellenberger *et al*., 2016). Briefly, soil samples were collected from various DuPont Pioneer‐owned properties in the USA. Bacterial colonies were isolated from soil samples by suspending 5 g of soil with 20 mL of phosphate‐buffered saline (PBS), spreading the diluted soil suspension onto various agar plates and incubating the plates at 26 °C for 2–4 days. Bacterial isolates were grown in tryptic soy broth (TSB, BD Diagnostic System, Germany) at 26 °C and 250 r.p.m. for 2 days. Cell pellets were obtained by centrifugation and washed once with PBS before storage at −80 °C. Total protein was extracted with B‐PER^™^ extraction buffer (Thermo Scientific, Waltham, MA, USA) with addition of a cocktail of several protease inhibitors (Catalog # 535141, Calbiochem, Billerica, MA, USA). Cleared cell lysates were used for screening in insect bioassays.

### Insect bioassays

Western corn rootworm (WCR, *Diabrotica virgifera virgifera*) bioassays were conducted in 96‐well plates by mixing 10 μL crude lysate with 50 μL diet in molten low‐melt agarose (Frontier Agricultural Sciences, Newark, DE) in each well. Three to five WCR neonates were placed into each well, and the assay plates were incubated at 25 °C for 4 days. Then, the insecticidal activity was scored as dead, severely stunted (survived with no growth), stunted (less than 60% growth to controls) or no activity (growth equal to controls). Extraction buffer was used as a negative control.

Quantitative bioassays were used to assess the potency of purified recombinant PIP‐47 proteins against rootworms with a two‐step diet incorporation protocol (Zhao *et al*., 2016). For WCR, ten concentrations ranging from 0.4 to 200 μg/mL in PBS were tested. For southern corn rootworm (SCR, *Diabrotica undecimpunctata howardi*) and northern corn rootworm (NCR*, Diabrotica barberi*), ten concentrations ranging from 1 to 750 μg/mL were tested. The total sample size per concentration was 60–64 for WCR and 30–32 for both NCR and SCR. Assay plates were incubated at 27 °C, 65 ± 5% RH for 9 days (WCR) and 7 days (NCR and SCR). Insects were scored as dead, severely stunted, stunted or not affected. The total numbers of dead and severely stunted larvae were used to calculate the growth inhibition concentrations affecting 50% of the larvae (IC_50_). Similarly, the mortality data for each bioassay were used to determine the lethal concentrations affecting 50% of larvae (LC_50_). Data for each bioassay were analysed following PROC PROBIT procedure using the C = option in SAS software (SAS Institute, 2013).

To check the activity of purified PIP‐47Aa toxin on other coleopteran, lepidopteran and hemipteran insects, we used previously described method (Schellenberger *et al*., 2016). Multiple doses of PIP‐47Aa toxin were tested, while sample buffer served as the negative control. Assays were conducted in four replicates per treatment, and 3–5 neonate larvae were manually infested in each replicate. There were 12–20 observations per treatment. Assays were scored for insect mortality and stunting of larval growth 4–5 days after infestation.

### DNA extraction and sequencing

Genomic DNA of SST62E1 was extracted from overnight culture with a Bacterial Genomic DNA Extraction Kit (Sigma‐Aldrich, St. Louis, MO, USA). The concentration of DNA was measured with a NanoDrop™ Spectrophotometer (Thermo Scientific, Waltham, MA, USA) and diluted to 40 ng/μL with sterile water. To amplify the 16S rDNA from SST62E1, a PCR (total in 25 μL) was set up by mixing 2 μL (40 ng/μL) genomic DNA preparation, 2 μL (5 μm) forward and reverse primers (TACCTTGTTACGACTT and AGAGTTTGATCMTGGCTCAG), 1 μL of 10 mM dNTP, 1× Phusion HF polymerase buffer and 1 unit of NEB Phusion High‐Fidelity DNA Polymerase (New England Biolabs, Ipswich, MA, USA). The PCR was run in a MJ Thermo Cycler (Model: PTC‐200 from Bio‐Rad Laboratories, Inc., Hercules, CA, USA) with a program including (i) 96 °C for 1 min; (ii) 30 cycles of 96 °C for 15 s, 52 °C for 2 min and 72 °C for 2 min; (iii) 72 °C for 10 min; and (iv) finally holding on 4 °C. QiaQuick^®^ DNA purification Kit was used to purify the PCR products (Qiagen, Hilden, Germany) and sequenced with Sanger sequencing method. Genomic DNA of SST62E1 was also generated using an Illumina™ library construction protocol and sequenced by a Genome Analyzer IIx (Illumina, San Diego, CA, USA). The protein open‐reading frames (ORFs) of minimum 100 nucleotides based on the assembled DNA contig sequences were predicted in six reading frames. All ORFs were annotated based on homology to sequences in the NCBI databases and stored in an internal database.

### Insecticidal protein purification and identification

Frozen SST62E1 cell pellets were resuspended in Buffer A (50 mM sodium acetate, pH 5) with 1× cocktail of protease inhibitors (Catalog # 535141 from Calbiochem). Cells were lysed by passing through a homogenizer (Cell Disrupter, Constant Systems Ltd., Kennesaw, GA, USA) at 30 000 psi, and a clear crude lysate, was obtained by a 20 min of 13 800 ***g*** centrifugation step. The lysate, adjusted with Buffer A to a conductivity of less than 5 mSiemens/cm was loaded onto a cation exchange column (SP‐HP HiTrap column from GE Healthcare, Pittsburgh, PA, USA), and the fractions were eluted with a linear salt gradient (0‐1 M NaCl) in Buffer A. Fractions were collected, desalted and then assayed for insect growth inhibition activity. Fractions shown insecticidal activity were combined, exchanged with Buffer B (50 mM Tris, pH 9) and loaded onto an anion exchange column (Mono Q™ column from GE Healthcare). Proteins were eluted with a salt gradient (0–1 M NaCl) in Buffer B. Active fractions, identified with bioassays, were subjected to further purification by size exclusion chromatography (Superdex 200 10/300 GL, GE Healthcare). Highly enriched, active fractions were analysed by SDS–PAGE. The candidate protein bands were excised and subjected to in‐gel trypsin digestion. The tryptic peptides were analysed by nanoscale liquid chromatography‐mass/mass spectrometry (Nano‐LC/ESI‐MS/MS) on a hybrid quadrupole Orbitrap™ mass spectrometer (Q Exactive™ from Thermo Fisher Scientific) coupled with an nano‐LC system (Eksigent^®^ Nano‐LC 1‐D Plus™ from AB Sciex, Framingham, MA, USA). The MS/MS spectra were collected in a data‐dependent acquisition (DDA) mode. Protein identification was carried out by database searches against a DuPont Pioneer in‐house database using Mascot search engine (Matrix Science Inc. Ltd., London UK).

### Recombinant protein expression and purification in *E. coli*

*PIP‐47Aa* gene was amplified from SST62E1 genomic DNA with a pair of forward and reverse primers (Table S3) and subcloned into an *E. coli* expression plasmid (pCOLD™‐1 from Takara Bio Inc., Kusatsu, Shiga, Japan) with an N‐terminal 6xHis tag followed by a Factor Xa cleavage site. The same approach was used to clone the genes of PIP‐47 homologs using the relevant genomic DNA and primer pairs (Table S3). These plasmid constructs were transformed into an *E. coli* competent cell line (BL21‐DE3) for expression of PIP‐47 proteins. Transformed cells were cultured at 37 °C overnight under selection of carbenicillin (100 μL/mL) and then diluted into fresh 2xYT medium (1 : 50) for continuous culturing. Once the cell density reached to about 0.8 OD, the cultures were cooled down to 16 °C. Protein inductions were achieved by the addition of 1 mM IPTG, and the cultures were continuously grown for 16 h at 16 °C. Recombinant proteins were isolated by Ni‐NTA agarose following the protocols from the manufacturer (Qiagen, Hilden, Germany).

### Corn expression vector construction and transformation

The corn expression cassette of PIP‐47Aa was constructed with the following elements: (i) an enhanced promoter consisting of three tandem copies of the enhancer element of the mirabilis mosaic virus (MMV) promoter (nucleotides number from 791 to 977 complement of GenBank accession # KT388099.1 (Dey and Maiti, 1999) linked to the sorghum promoter Sb‐RCc3 (SEQ ID NO. 1 in patent application WO 2012112411 A1); (ii) a plastid targeting sequence from the maize chlorophyll *a*‐*b* binding protein (nucleotides number from 707 to 910 complement of GenBank accession # NM_001155421.1) fused in frame to the *PIP‐47Aa* gene; and (3) a transcriptional terminator (Pin II) from *Solanum tuberosum* (An *et al*., 1989). The *PIP‐47Aa* expression cassette was then subcloned into a binary plant transformation vector backbone by Gateway™‐mediated recombination (Thermo Fisher Scientific). The resultant vector ZmPIP‐47Aa contains the *PIP‐47Aa* cassette upstream of the selectable marker gene, phosphomannose isomerase (PMI) (Reed *et al*., 2001), driven by the maize Ubiquitin promoter, 5′UTR and intron (Christensen *et al*., 1992). *Agrobacterium*‐mediated stable maize transformation was performed by the method of Cho *et al*. (2014) using PMI with mannose selection in the commercial maize elite‐inbred line PHR03. Regenerative green tissues were transferred to PHI‐XM medium with mannose selection. Shoots were transferred to tubes containing MSB rooting medium for rooting and plantlets transplanted to soil in pots in the glasshouse.

### In‐planta efficacy assessment

T0 plants transformed with vector ZmPIP‐47Aa were grown in the glasshouse under standard conditions. After a period of approximately 18 days, plants were transplanted into 3.5‐L plastic pots with Fafard superfine germinating mix and 1 tsp Osmocote (The Scotts Company, Marysville, OH, USA) added in approximately the middle layer of each pot. Plants were watered to maintain moderate soil moisture and fertilized daily with Peters Excel 15‐5‐15 Cal‐Mag Special Fertilizer (Everris NA Inc., Dublin, OH, USA) at a rate of ~75 μg/mL. Nondiapausing WCR eggs derived from a colony maintained by the DuPont Pioneer Insect Production Research Group (Johnston, IA) were washed and suspended in a 0.8% agar solution. Two hundred eggs were pipetted into the soil near each plant at approximately stage V3, and another two hundred eggs were applied one week later. Approximately 20 days after the first infestation, plant roots were washed and scored using the method developed by researchers at Iowa State University (Oleson *et al*., 2005). Data were analysed using one‐way ANOVA using JMP (JMP 13, SAS Institute, Cary, NC, USA).

### In‐planta expression evaluation of T0 events expressing PIP‐47Aa

Corn root tissues from T0 plants were lyophilized and pulverized. Six to seven milligrams of each sample was suspended in 350 μL phosphate‐buffered saline with Tween‐20 (PBST) containing cOmplete™ Proteinase Inhibitor/EDTA‐free (Roche Diagnostics Corporation, Indianapolis, IN, USA). After sonication for 2 min, followed by centrifugation at 4 °C, 20 000 ***g*** for 15 min, the supernatant was transferred to new tubes and 5–10 μL was used to determine protein concentrations using BCA assay (Thermo Fisher Scientific). A mixture of 120 μL supernatant, 40 μL 4× LDS, 4% β‐ME with protease inhibitor was heated at 80 °C for 10 min and clarified by centrifugation. Eight microlitres of clear supernatant was added in wells of Bis‐Tris Midi gels (4%–12%) (Thermo Fisher Scientific) with MES as the running buffer for protein separation by electrophoresis. An iBlot apparatus (Invitrogen, Carlsbad, CA, USA) was used to transfer the protein bands from the gel onto a nitrocellulose membrane. The membrane was first incubated in PBST with skim milk powder at 5% for 2 h. After addition of affinity‐purified polyclonal anti‐PIP‐47Aa antibody generated from rabbit at 1 : 20 000 dilutions in PBST, the membrane was further incubated for overnight. After rinsing with PBST three times, the membrane was then incubated with goat anti‐rabbit‐HRP (Bio‐Rad Laboratories) in PBST for 3 hr. The presence of the antibody bound PIP‐47Aa protein was captured by a Fujifilm Imager (Fujifilm, Minato, Tokyo, Japan).

### Testing for cross‐resistance to mCry3A

A WCR colony resistant to mCry3A (Zhao *et al*., 2016) was used to evaluate the cross‐resistance of PIP‐47Aa. Purified PIP‐47Aa in nine concentrations ranging from 4.38 to 280 μg/mL in Tris‐buffered saline (TBS) buffer was tested against both mCry3A‐susceptible and mCry3A‐resistant strains. Buffer alone was included as a negative control. The mixture of 15 μL PIP‐47Aa solution and 65 μL diet was added into each well of 96‐well plate to reach the target concentration. Plates were allowed to air dry, and three larvae (<24 h post hatching) were added per well. The plates with WCR larvae were incubated at 27 °C, 65% relative humidity in dark for 3 days. The plates were scored as dead or severely stunted (>60% of growth reduction compared to buffer control) based on the least affected individual for each well. The background mortality from buffer control was <5% in all bioassays. There were a total of 15–24 observations for each concentration, with 7–8 wells per replicate and 2–3 replicates per treatment. The mortality data for each WCR strain were used to obtain the LC_50_, and the total numbers of dead and severely stunted larvae were used to obtain the IC_50_. All data were analysed using PROC PROBIT in SAS (2013). The insect resistance ratio (RR) was calculated using the formula listed below:

$$\begin{array}{cc} \text{RR} & =({\text{LC}}_{50}\;\text{or}\;{\text{IC}}_{50}\;\text{value of resistant WCR})\\ & /({\text{LC}}_{50}\;\text{or}\;{\text{IC}}_{50}\;\text{value of susceptible WCR}). \end{array}$$

### Brush border membrane vesicle (BBMV) preparation and BBMV binding assays

Midguts were extracted from third instars of laboratory‐maintained nondiapausing WCR (Pioneer insectary, Johnston, IA). Removal of the entire gut was achieved by grasping larvae just behind the head capsule with forceps while holding the anal plate region with another forceps and pulling. Extracted guts were cleaned of surface fat bodies and connective tissue and immediately flash‐frozen in liquid nitrogen for storage at −80 °C until needed. BBMVs were prepared from frozen midgut tissue as described essentially by Wolfersberger *et al*. (1987). Determinations of proteins were performed using BCA Kit (Thermo Fisher Scientific, Waltham MA, USA). Enrichment of brush border apical membrane in BBMV preparations was typically sixfold to eightfold and was assessed by measuring and comparing the aminopeptidase activity (enzyme associated with the apical membrane) in the final BBMV suspension to the activity in the initial crude midgut homogenate. Aminopeptidase enzymatic activity was determined by measuring the rate of hydrolysis of the artificial substrate 1 mM l‐leucine‐*p*‐nitroanilide (Sigma) in 25 mM NaCl, 10 mM Tris–HCl, pH 8.0, while monitoring absorbance at 405 nm in a 96‐well plate reader Flexstation 3 (Molecular Devices, Sunnyvale, CA, USA).

Specific binding of PIP‐47Aa was established using a standard PBST buffer containing a protease inhibitor cocktail (cOmplete™ EDTA‐free Protease Inhibitors, Roche). Competition binding experiments utilized a buffer that provided favourable solubility conditions for PIP‐47Aa and the competitors (IP3‐H9 and AfIP‐1A/1B). That buffer consisted of 50 mM NaCl, 2.7 mM KCl, 8.1 mM Na_2_HPO_4_, 1.47 mM KH_2_PO_4_, pH 7.5, supplemented with 0.1% Tween‐20, and protease inhibitor cocktail (Roche). Prior to binding experiments, proteins were quantified by gel densitometry following Simply Blue^®^ (Thermo Scientific) staining of SDS–PAGE resolved samples that included BSA as a standard. To track binding, proteins were labelled with Alexafluor 488 (Life Technologies, Carlsbad, CA, USA) according to manufacturer's recommendations. To evaluate specific binding, BBMVs were incubated in binding buffer (100 μL) with Alexa‐labelled toxin for 1 h at ambient temperature with constant agitation on a high velocity orbital shaker. The binding reaction was terminated by the addition of 1 ml fresh binding buffer followed by centrifugation at 13 000 ***g*** for 10 min at 4 °C. The resulting pellet was washed twice by resuspending in 0.5 mL binding buffer followed by centrifugation. The final binding pellets were solubilized in 20 μL sample buffer (Novex LDS, Invitrogen), boiled for 5 min and resolved with SDS–PAGE (NuPage^®^ 4%–12% Bis‐Tris, Invitrogen). To measure nonspecific binding, the same binding reaction was tested in parallel with the addition of a saturating concentration of unlabelled toxin that was determined empirically by homologous competition binding assays. Residual binding of Alexa‐labelled proteins was detected in‐gel using a digital fluorescence imaging system (LAS4010, GE Healthcare) and quantified by densitometry software (Phoretix 1D, TotalLab Ltd., Newcastle upon Tyne, UK).

## Author contributions

J.‐Z.W., L.L., U.S., A.L.L., M.E.N., G.W. designed experiments. J.O., B.A.R., Y.J.P., M.J.M., G.Z., W.X., L.P., C.P.O., J.Z.Z., N.Y., V.C.C., S.H.D., G.A.S. performed the experiments. L.L., A.K., N.Y., J.Z.Z, M.E.N., J.Z.W. analysed the data. J.‐Z.W, L.L., A.L.L., N.Y., M.E.N., G.W. wrote the manuscript.

## Supporting information


**Table S1** Homologous proteins of PIP‐47Aa derived from microbial genomes
**Table S2** Sequence identities of homologous PIP‐47 proteins
**Table S3** Cloning primers for *PIP‐47* homologs for expression in *E. coli*
