# Supplementary material for: A selective insecticidal protein from Pseudomonas mosselii for corn rootworm control
Source: Plant Biotechnol J. 2017 Oct 1;16(2):649–59. doi: 10.1111/pbi.12806 (PMC5787824; doi:10.1111/pbi.12806)
Supplement: Supplementary file 1 — Table S1 Homologous proteins of PIP‐47Aa derived from microbial genomes Table S2 Sequence identities of homologous PIP‐47 proteins Table S3 Cloning primers for PIP‐47 homologs for expression in E. coli [file PBI-16-649-s001.docx]

**Table S1** Sources of PIP-47Aa and homologs

| **Protein** | **Identity to PIP-47Aa** | **Source** | **Species** | | **NCBI accession number** | |  |
| --- | --- | --- | --- | --- | --- | --- | --- |
| PIP-47Aa | 100.0% | DuPont Pioneer Collection - SST62E1 | *Pseudomonas mosselii* | | KY982916 | |  |
| PIP-47Ab | 99.7% | DuPont Pioneer pooled sample - XM31 | N/A | | KY982914 | |  |
| PIP-47Ba | 83.5% | DuPont Pioneer Collection - N1E2; | *Pseudomonas entomophila* | | KY982917 | |  |
| PIP-47Bb | 88.0% | DuPont Pioneer Collection - SSP454B4-1 | *Pseudomonas oryzihabitans* | | KY982918 | |  |
| PIP-47Bc | 88.8% | DuPont Pioneer Collection - PMC3030C3-1 | | *Pseudomonas monteilii* | | KY982919 | |
| PIP-47Fa | 48.5% | DuPont Pioneer Collection - SSP459B9-3 | *Pseudomonas chlororaphis* | | KY982920 | |  |
| PIP-47Fb | 44.0% | DuPont Pioneer pooled sample - HK5 | N/A | | KY982915 | |  |
| PIP-47Ga | 32.8% | NCBI database | *Photorhabdus luminescens* | | WP_011146303 | |  |
| PIP-47Gb | 37.0% | NCBI database | *Photorhabdus luminescens* | | WP_011146302 | |  |
| PIP-47Gc | 38.4% | NCBI database | *Photorhabdus asymbiotica* | | WP_015834697 | |  |
| PIP-47Gd | 36.4% | NCBI database | *Photorhabdus asymbiotica* | | WP_015834680 | |  |
| PIP-47Ge | 35.8% | NCBI database | *Klebsiella oxytoca* | | ZP_17107944 | |  |
| PIP-47Gf | 39.5% | NCBI database | *Vibrio metschnikovii* | | ZP_05881874 | |  |
| PIP-47Gg | 39.6% | NCBI database | *Vibrio Fluvialis* | | WP_020327647 | |  |
| PIP-47Gh | 35.8% | NCBI database | *Raoultella ornithinolytica* | | KAJ96469 | |  |

**Table S2** Identities of PIP-47Aa and homologs

|  | **PIP-47Ab** | **PIP-47Ba** | **PIP-47Bb** | **PIP-47Bc** | **PIP-47Fa** | **PIP-47Fb** | **PIP-47Ga** | **PIP-47Gb** | **PIP-47Gc** | **PIP-47Gd** | **PIP-47Ge** | **PIP-47Gf** | **PIP-47Gg** | **PIP-47Gh** |
| --- | --- | --- | --- | --- | --- | --- | --- | --- | --- | --- | --- | --- | --- | --- |
| **PIP-47Aa** | 99.7 | 84.1 | 88.5 | 88.8 | 44.7 | 38.5 | 34.6 | 38.3 | 40.5 | 36.1 | 36.6 | 38.9 | 40.5 | 36.7 |
| **PIP-47Ab** | - | 84.1 | 88.5 | 88.8 | 44.7 | 38.5 | 34.6 | 38.3 | 40.5 | 36.1 | 36.6 | 40.7 | 40.5 | 36.7 |
| **PIP-47Ba** | - | - | 81.4 | 81.4 | 42.8 | 39.1 | 35.1 | 40.7 | 40 | 39.9 | 39.7 | 40.7 | 40.1 | 39.3 |
| **PIP-47Bb** | - | - | - | 99.7 | 45.3 | 42.4 | 36.4 | 41.3 | 40.9 | 38.8 | 41.6 | 40.2 | 41.4 | 40.2 |
| **PIP-47Bc** | - | - | - | - | 45.3 | 42.4 | 36 | 41.3 | 40.9 | 38.8 | 41.6 | 40.2 | 41.4 | 40.2 |
| **PIP-47Fa** | - | - | - | - | - | 40.2 | 32.3 | 34.8 | 36.3 | 31.7 | 39.2 | 37.7 | 38.5 | 39.9 |
| **PIP-47Fb** | - | - | - | - | - | - | 35.8 | 38.6 | 39.4 | 37.6 | 86.8 | 47.5 | 49.6 | 87.6 |
| **PIP-47Ga** | - | - | - | - | - | - | - | 47.1 | 45 | 73.9 | 39.2 | 34.7 | 35.8 | 38.5 |
| **PIP-47Gb** | - | - | - | - | - | - | - | - | 88.6 | 50 | 39.6 | 41.4 | 39.2 | 38 |
| **PIP-47Gc** | - | - | - | - | - | - | - | - | - | 48.6 | 40 | 41.1 | 39.6 | 39.6 |
| **PIP-47Gd** | - | - | - | - | - | - | - | - | - | - | 39.4 | 36.4 | 37 | 38.7 |
| **PIP-47Ge** | - | - | - | - | - | - | - | - | - | - | - | 48.6 | 49.8 | 98.9 |
| **PIP-47Gf** | - | - | - | - | - | - | - | - | - | - | - | - | 83.8 | 48.9 |
| **PIP-47Gg** | - | - | - | - | - | - | - | - | - | - | - | - | - | 49.6 |
| **PIP-47Gh** | - | - | - | - | - | - | - | - | - | - | - | - | - | - |

**Table S3** Cloning primers for *PIP-47* homologs for expression in *E. coli*.

| **Gene** | **Forward primer** | **Reverse primer** |
| --- | --- | --- |
| *PIP-47Aa* | AATCATATGCACGCTCCAGGAGCAATTCCATCG | CAGGATCCTCAGCTGACCAGGCAGATCGCC |
| *PIP-47Ba* | AATCATATGCACGCTCCAGGAGCCGCCCCATCC | CAGGATCCTCAGCTGACCAGGCAGATTGCCCG |
| *PIP-47Bb* | AATTAGCATGCATATGCACGCACCAACAGTGAAAGAACTCGCCCAC | ACAGGATCCTCAGCCGACCAGGCAGATTGCCCGCAGC |
| *PIP-47Fa* | AATTACATATGAGCACTCAAAATCACAAGCACATTAC | AATTAGATCTCTATTGACCCTCTAAGCAAGTCGCTCTTG |
| *PIP-47Ga* | This gene was synthesized based on the sequence from NCBI. | |
